# Aging Associated Transcriptomic Signatures in Tumor and Tumor Adjacent Lung Tissues Associated with Recurrence Following Resection of Stage I Lung Adenocarcinoma

**DOI:** 10.21203/rs.3.rs-8971302/v1

**Published:** 2026-03-08

**Authors:** Fares Darawshy, Cigdem Sevim Bayrak, Xianxiao Zhou, Kendrew Wong, Imran Sulaiman, Benjamin Kwok, Cecilia Chung, Alena Lukovnikova, Sofia Roldan, Benjamin G. Wu, Matthias C. Kugler, Yonghua Li, Rosemary Schluger, Destiny Collazo, Yaa Kyeremateng, Ray Pillai, Matthew Blaisdsell, Michelle Fridman, Alexander Bain, Marcus D. Goncalves, Chandra Goparaju, Daniel Sterman, Anil Vachani, Gregory David, Aristotelis Tsirigos, Bin Zhang, Christian V. Forst, Harvey Pass, Leopoldo N. Segal, Jun-Chieh J. Tsay

**Affiliations:** New York University Grossman School of Medicine, NYU Langone Health; Icahn School of Medicine at Mount Sinai; Icahn School of Medicine at Mount Sinai; New York University Grossman School of Medicine, NYU Langone Health; New York University Grossman School of Medicine, NYU Langone Health; New York University Grossman School of Medicine, NYU Langone Health; New York University Grossman School of Medicine, NYU Langone Health; New York University Grossman School of Medicine, NYU Langone Health; New York University Grossman School of Medicine, NYU Langone Health; New York University Grossman School of Medicine, NYU Langone Health; New York University Grossman School of Medicine, NYU Langone Health; New York University Grossman School of Medicine, NYU Langone Health; New York University Grossman School of Medicine, NYU Langone Health; New York University Grossman School of Medicine, NYU Langone Health; New York University Grossman School of Medicine, NYU Langone Health; New York University Grossman School of Medicine, NYU Langone Health; New York University Grossman School of Medicine, NYU Langone Health; New York University Grossman School of Medicine, NYU Langone Health; New York University Grossman School of Medicine, NYU Langone Health; New York University Grossman School of Medicine, NYU Langone Health; NYU School of Medicine; New York University Grossman School of Medicine, NYU Langone Health; New York University Grossman School of Medicine, NYU Langone Health; New York University Grossman School of Medicine, NYU Langone Health; NYU Grossman School of Medicine; Icahn School of Medicine at Mount Sinai; Icahn School of Medicine at Mount Sinai; NYU School of Medicine; New York University Grossman School of Medicine, NYU Langone Health; New York University Grossman School of Medicine, NYU Langone Health

**Keywords:** lung cancer, recurrence, transcriptome, aging, mortality

## Abstract

**Background:**

Age is an independent prognostic factor in early-stage non-small cell lung cancer (NSCLC), yet the molecular differences between old and young patients and their contribution to disease progression remain unclear. We investigated age-related transcriptomic differences in early-stage lung adenocarcinoma (LUAD) and their association with recurrence.

**Methods:**

Tumor and adjacent normal lung tissue (NAT) from 126 stage I LUAD patients underwent bulk RNA sequencing to characterize age-related transcriptomic profiles. Differential expression and multiscale embedded gene co-expression network analysis (MEGENA) were used to identify age- and recurrence-associated modules. Pathways were annotated using Ingenuity Pathway Analysis. External confirmation was performed using TCGA (n=256) and TRACERx (n=83) cohorts.

**Results:**

Based on the cohort’s median age, 60 patients were classified as old (>70 years) and 66 as young (≤70 years). In tumors, older patients with recurrence showed marked upregulation of cancer-associated, inflammatory, and extracellular matrix pathways compared with older patients without recurrence. In NAT samples, older patients with recurrence demonstrated upregulation of inflammatory and cancer-associated pathways—including phagosome formation, IL-17, IL-6, and Th2 signaling—that were absent or downregulated in young patients. MEGENA revealed a larger number of recurrence-associated co-expression modules in old versus young patients. These age-related patterns were highly conserved in both external cohorts across tumor and NAT samples.

**Conclusion:**

Aging in LUAD is associated with distinct cancer- and inflammation-related transcriptomic alterations that contribute to recurrence. Aging-related molecular signatures may improve risk stratification for early-stage lung cancer.

**Impact:**

Aging shapes tumor microenvironment transcriptomes in stage I LUAD, enabling improved relapse risk stratification after surgery

## Introduction

Non-small cell lung cancer (NSCLC) is a leading cause of cancer-related morbidity and mortality worldwide^1^. The highest incidence and increasing prevalence are now observed in the elderly population, with age recognized as an independent prognostic factor influencing survival^2–4^. However, the biological mechanisms by which aging contributes to NSCLC progression are not fully elucidated.

Aging induces various molecular alterations, including immune landscape remolding and gene expression changes, that might affect lung cancer progression and increase mortality^5^. Increased inflammatory signaling and the accumulation of senescent cells characterize the aging immune environment^6^. These senescent cells can secrete pro-tumorigenic factors, known as senescence-associated secretory phenotype (SASP), promoting tumor progression^6–9^. Alteration in gene expression is another aging-related change that can potentially act as tumor regulators or promote tumor growth, ultimately leading to poor outcomes.

Recent studies have highlighted age-specific genomic and transcriptomic changes in NSCLC that are associated with survival. Cai et al. reported an age-dependent trend in genomic alterations, highlighting an increase in tumor mutation burden and the number of mutations such as KRAS, MET, and PIK3CA, alongside a decrease in ALK and EGFR mutations^10^. Another study found aging and senescence-induced genes upregulation in lung cancer tissues compared to healthy controls^11^. An investigation used transcriptomic profiles from 2,518 patients across twelve NSCLC cohorts to construct an aging-related risk score associated with increased infiltration of immune suppressive cells, which could be used to predict cancer prognosis and response to immunotherapy^12,13^. Despite these findings, several confounders influence these studies, such as the inclusion of varying histology, disease stages, and treatment modalities, which may confound the specific impact of aging on NSCLC outcomes. Furthermore, conflicting statistics regarding age and disease outcome have been reported in NSCLC^14^; despite that chronological age correlates with worse overall survival, in models adjusted for treatment intensity and comorbidities, age was not highly associated with cancer-specific outcomes^15,16^. Therefore, a better understanding of the relationship between aging and cancer is needed.

In this study, we leveraged an internal cohort and two independent external cohorts to evaluate tissue-specific gene expression signatures associated with age and prognosis, focusing on early-stage (stage I) lung adenocarcinoma (LUAD) treated by surgical resection, while minimizing confounding variables. Overall, our data demonstrate that poor prognosis, defined by post-surgical tumor recurrence, among elders was characterized by cancer- and inflammation-related alterations in gene expression.

## Methods

### Subjects and samples collection

Tumor and normal adjacent lung tissue (NAT) were obtained from 126 patients with stage I lung adenocarcinoma (LUAD) who underwent surgical resection at the NYU Langone Medical Center between the years 2006–2015. Patients identified on preoperative workup as having a lung nodule suspicious of lung cancer were recruited into our NYU Lung Cancer Biomarker Cohort. Sample collection and processing followed our Early Detection Research Network (EDRN) approved standard operating procedures based on the principles of prospective-specimen-collection, retrospective-blinded evaluation (PRoBE) study design^17^. Samples used for these investigations were snap frozen under sterile conditions in the operating room and then stored at −80°C until use.

The subject’s demographic, clinical information, and outcomes were prospectively collected in a secure Research Electronic Data Capture (REDCap) database. Subjects included in this investigation completed at least 5 years of follow-up post-surgery, with CT scans performed for surveillance to document any systemic or locoregional recurrences. This study was approved by the New York Grossman University School of Medicine Institution Review Board (IRB 8896). Written informed consent was obtained from all patients. The study was conducted in accordance with the recognized ethical guidelines of Declaration of Helsinki. Patients were defined as old (> 70 years old) and young (≤ 70 years old) based on median age (70 years) of our NYU internal cohort. Notably, this threshold is close to the reported median age at lung cancer diagnosis in the general population (71 years), supporting its biological and clinical relevance^4,18^. Recurrence was defined as pathological confirmation of any loco-regional or systemic recurrence of NSCLC during their five years post-surgical follow-up.

#### External cohorts

Publicly available data from two independent cohorts of stage I LUAD were used for comparison. The cancer genome atlas (TCGA) data generated by the TCGA Research Network (https://www.cancer.gov/tcga), and TRACERx^19^. We included tumor and normal adjacent tissue samples from stage I LUAD patients who underwent surgical resection with gene expression profiles and clinical data. Samples without transcriptome data, patients without follow-up information or recurrence with a second primary cancer were excluded from analysis. TCGA data were obtained using the TCGAbiolinks *R* package and cBioportal.org^20–22^. TRACERx data were downloaded from the original publication^19^. External cohorts’ analysis followed a similar structure to the NYU cohort, using the same age cutoff of 70 years to define patients as old or young. Where some patients had multiple tumor samples profiled, raw gene-level counts were summed across samples from the same patient to generate a single aggregated expression profile per individual, ensuring that each patient contributed only one observation to downstream analyses.

#### Transcriptomic gene expression

RNA sequencing was performed on frozen surgical samples from both tumor and NAT in the NYU internal cohort. RNA was extracted using RNeasy Plus Mini kit (Qiagen); the total input RNA for library prep was 10ng. Libraries were prepared using Nugen Trio RNA-Seq with AnyDeplet-Human (Tecan). Libraries were then sequenced using NovaSeq 6000 (S2 100 cycle Flowcell). Processed genes count tables were used for analyses of TCGA and TRACERx transcriptomic data. Resulting count data were used for further analysis in *R* (version 4.1.0) with vegan (version 2.6–2), and tidyverse (version 1.3.2) packages. Analysis was performed comparing old vs. young patients and clinical outcome (recurrence) within each group. Permutational multivariate analysis of variance (PERMANOVA) was used to examine differences in overall transcriptomic composition. To evaluate gene expression differences between old and young patients and between recurrence groups, we used the edgeR Bioconductor package (version 3.36.0)^23^. Functional pathway analysis using differentially regulated genes (False Discovery Rate - FDR < 0.2) was done using QIAGEN IPA (QIAGEN Inc., https://digitalinsights.qiagen.com/IPA)^24^. This permissive cutoff was selected given the exploratory nature of aging-associated transcriptomic profiling, the emphasis on pathway-level inference rather than single-gene discovery, and our goal seeking to compare transcriptomic signatures present in three independent different datasets, consistent with what has been done in prior studies^25–27^. Additional models adjusting were performed for common confounders (sex, stage and smoking status). Models adjusting for pathological features (only partially available in the three different cohorts) were explored but were restricted to a substantially smaller annotated subset, resulting in marked reduction in statistical power; therefore, only core adjusted models were retained as the primary analyses. Gene Set Enrichment Analysis (GSEA) was performed using significantly dysregulated differential genes (FDR < 0.2) to compare consistency across cohorts, using the fgsea package^28^.

#### Gene Co-expression Network

To further investigate the overlap of transcriptomic signatures among the RNA-seq datasets of the NYU, TCGA and TRACERx cohorts related to different samples and outcomes, a gene co-expression network analysis using MEGENA^29^ was applied. Prior to network construction, genes with low or zero expression were filtered out, and log_2_-transformed expression values were standardized within each dataset using Z-transformation. We identified 15,681 genes common across all three cohorts and merged the datasets accordingly. To correct for potential batch effects due to dataset origin, we applied a linear model to remove cohort-specific variation, and the resulting residuals were used as input for co-expression analysis. Co-expression networks were subsequently built by MEGENA, incorporating all samples from the three cohorts to identify modules relevant to shared transcriptomic features. Specifically, gene-gene co-expression relationships were calculated using Pearson’s correlation, and the resulting network was filtered using the Planar Filtered Network (PFN) algorithm to retain significant gene co-expression relationships. Subsequently, the PFN underwent an unsupervised multi-scale clustering analysis and hub analysis to identify gene co-expression subnetwork clusters (hereafter modules). Modules were compared to the distribution of random modules of 100 permutations to calculate module significance. Significant modules and hubs were identified at FDR < 0.05 at optimized resolution parameters. Modules were ranked based on significant enrichment for differentially expressed gene (DEG) signatures from the NYU cohort and identified separately for each age group (≤ 70 and > 70 years) in both tumor and NAT samples. These modules were then compared between recurrence groups to identify those associated with age or recurrence. The modules significantly enriched with DEG were further evaluated in the TCGA and TRACERx cohorts to determine their conservation across cohorts. To annotate the pathway functions of network modules, we performed functional enrichment analysis based on Fisher’s Exact Test^30^, as well as quantitative Gene Set Enrichment Analysis (GSEA)^31^ to determine what the module genes were enriched for Gene Ontology (GO) biological processes. We adjusted the multi-testing p-values using the Benjamini-Hochberg (BH) procedure^32^. Adjusted *p*-value < 0.05 was considered significant.

#### Statistical analysis and Data availability

Continuous variables are reported using median and interquartile range (IQR), while categorical variables are reported as number and percentage [N (%)]. To compare continuous variables between two groups, we used *chi*-square test, while Wilcoxon Rank-Sum test (Mann-Whitney U test) was used to compare categorical variables. Recurrence rate compared between old and young patients was performed using the Kaplan-Meier curve and log rank test. Raw data of the NYU cohort from the RNA sequencing is available at Sequence Read Archive PRJNA987649. TRACERx annotated tables are available at https://doi.org/10.5281/zenodo.7603386^19^. The annotated tables and analytical codes used for the analyses presented in the current manuscript are available at https://github.com/segalmicrobiomelab/aging.lung.transcriptome.lung.cancer.git

## Results

### Patients’ characteristics

One hundred twenty-six early-stage LUAD patients were grouped into old (n = 60) and young (n = 66) based on the median age of the NYU cohort (70.0 years). Table 1 shows the demographics and clinical characteristics of these patients, which were composed of 30% males, 80.2% white, and 73.8% ever smokers. Unsurprisingly, older patients had a higher 5-year mortality rate than younger patients (20% vs. 4.5%, p = 0.01), but no significant difference in recurrence rate (23.3% vs. 19.7%, ns, **Supplementary Fig. 1**). There was also no difference between the type of recurrence (systemic vs. locoregional).

To compare and validate our age-related transcriptomic signatures associated with recurrence, we performed a similar analysis in the TCGA cohort (n = 256) that included 256 tumor samples and 29 NAT samples, and in the TRACERx cohort (n = 83) that included 83 aggregated tumor samples. **Supplementary Table 1** shows characteristics of patients with stage I LUAD in both cohorts. Both cohorts were slightly younger than the NYU cohort, with a median age of 67 and 68, respectively. Similar to the NYU cohort, 5-year mortality rates among old patients were higher than young patients in both TCGA and TRACERx (p = 0.01 and p = 0.006, respectively). Kaplan-Meier curves showed no significant differences in recurrence between old and young in TCGA, while in TRACERx, recurrence was significantly higher in old patients (**Supplementary Fig. 2A, 2B)**.

#### Tumor and NAT transcriptomics differences in old and young NSCLC patients

RNA-seq data were obtained on 126 tumor and 126 paired NAT samples from 126 patients with stage I LUAD in the NYU Internal cohort. PCoA analyses based on the Bray-Curtis Dissimilarity Index showed no differences in transcriptomic composition between old and young patients in both tumor and NAT (**Supplementary Figs. 3A, C**, PERMANOVA, p = ns). Differential gene expression using edgeR analysis identified 662 genes significantly upregulated, and 347 genes downregulated when comparing old to young patients, in tumor samples (**Supplementary Figs. 3B)**. Among NAT samples, 2685 significantly upregulated and 874 downregulated genes were associated with aging (**Supplementary Figs. 3B, 3D, Supplementary Table 2**). We then utilized Ingenuity Pathway Analysis (IPA) to summarize pathways associated with aging based on differentially expressed genes (**Supplementary Fig. 4)**. Overall, while there is some concordance of pathways identified as associated with aging in tumor and NAT, we identified more upregulated pathways in NAT samples. These pathways were related to cancer, aging, and inflammation-related, such as Cell Cycle related pathways, Interferon-gamma, Tumor microenvironment, IL-17, and IL-6 pathways (**Supplementary Table 3**).

In the TCGA cohort, beta-diversity analyses also showed no statistically significant transcriptomic compositional differences between old and young patients in tumor or NAT samples (**Supplementary Figs. 5A, 5C**). edgeR analysis identified 1228 upregulated genes and 2235 downregulated genes when comparing old to young patients, in tumor samples. Among NAT samples, aging was associated with upregulation of 168 and downregulation of 24 genes (**Supplementary Figs. 5B, 5D, Supplementary Table 4**). Similar to the NYU cohort, pathways associated with aging in tumor and NAT samples included upregulation of pathways related to extracellular matrix and inflammatory pathways such as phagosome formation, IL-4, and IL-13 (**Supplementary Fig. 5E, Supplementary Table 5**). In TRACERx tumor samples, beta-diversity analyses showed significant compositional differences between old and young (**Supplementary Fig. 6A**, PERMANOVA; p = 0.01), and 1918 genes were significantly upregulated and 1206 downregulated among the older patients (**Supplementary Fig. 6B, Supplementary Table 4**). This was annotated to the following top pathways: regulation of IGF, extracellular matrix (ECM) organization, and phagosome formation (**Supplementary Fig. 6C, Supplementary Table 5**). Thus, multiple transcriptomic differences were noted between old vs. young patients in their tumor and NAT samples with a common pattern of increased activity of pathways linked to inflammation, immune signaling, and cancer progression in older patients.

Differential gene expression analyses were repeated while adjusting for common confounders (sex, smoking status and stage). Results were concordant between unadjusted and core-adjusted models across tumor and NAT tissues showing pathway-level enrichment patterns were similarly preserved after adjustment (**Supplementary Figs. 7–8, Supplementary Tables 6–7**).

### Tumor transcriptomic signatures associated with recurrence vary between age groups

**Figure 1:**
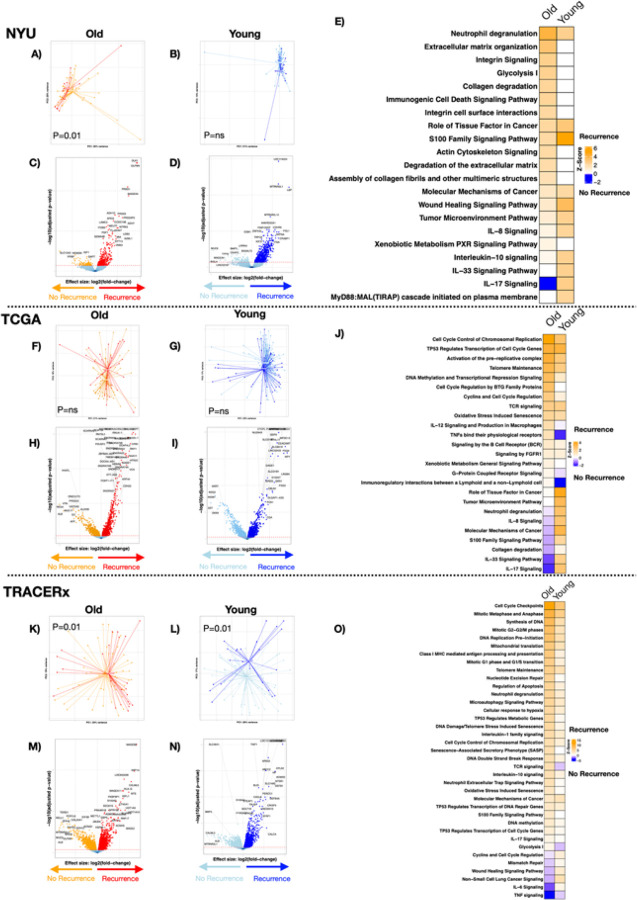
Transcriptomic signatures associated with recurrence in tumor tissue. PCoA of beta diversity (Bray-Curtis) based on recurrence showed significant differences in A) old, but not in B) young patients in the NYU cohort. P-value by PERMANOVA. C, D) volcano plot of differentially expressed genes (FDR <0.2) between subjects with recurrence and without recurrence in old and young patients in the NYU cohort, respectively. E) heatmap of canonical pathway analysis based on Ingenuity Pathway Analysis (IPA) comparing recurrence vs. no recurrence groups in old and young patients within the NYU cohort. Yellow shows upregulation of pathways, and blue shows downregulation. F, G) PCoA of TCGA cohort showing the differences in beta diversity between subjects with and without recurrence among old and young patients, respectively (p=ns, PERMANOVA). H, I) TCGA cohort volcano plot of differentially expressed genes (FDR <0.2) between subjects with recurrence and without recurrence in old and young patients, respectively. J) TCGA cohort, heatmap of canonical pathway analysis based on IPA comparing recurrence vs. no recurrence in old and young patients. Yellow shows upregulation of pathways, and blue shows downregulation. K, L) PCoA of TRACERx cohort showing the differences in beta diversity between subjects with and without recurrence among old and young patients, respectively (p=0.01 and p=0.01, PERMANOVA). M, N) TRACERx cohort volcano plot of differentially expressed genes (FDR <0.2) between subjects with recurrence and without recurrence in old and young patients, respectively. O) TRACERx cohort, heatmap of canonical pathway analysis based on IPA comparing recurrence vs. no recurrence in old and young patients. Yellow shows upregulation of pathways, and blue shows downregulation.

We evaluated tumor samples from the NYU cohort for transcriptomic signatures associated with recurrence, in old and young patients separately. Beta-diversity analyses showed statistically significant transcriptomic compositional differences between patients with recurrence and those without cancer recurrence within the older group, but not the young group (Figs. 1A, 1B PERMANOVA, p = 0.01, p = ns, respectively). edgeR analysis found that in tumor samples from old patients, there were 410 genes upregulated, and 114 genes downregulated associated with recurrence; while in young patients, there were 718 genes upregulated, and 131 genes downregulated associated with recurrence (Figs. 1C, 1D, **Supplementary Table 8**). Figure 1E shows the top dysregulated pathways associated with tumor recurrence in old and young patients. Among the top pathways upregulated in the tumor samples from old patients with recurrence, we identified pathways related to neutrophilic degranulation, ECM organization and glycolysis (**Supplementary Table 9**), while other inflammatory pathways seemed to be more upregulated among the young group, such as IL-17 signaling.

Next, we evaluated tumor transcriptomic signatures associated with recurrence among old and young groups in TCGA and TRACERx cohorts. In TCGA tumor samples, beta-diversity analyses showed no statistically significant differences between recurrence groups (Figs. 1F, G, PERMANOVA, p = ns). Among the older patients’ group, 1,413 genes were significantly upregulated and 913 downregulated among those with recurrence; and in young patients, 2,779 upregulated and 1,697 downregulated (Figs. 1H, I, **Supplementary Table 10**). Pathway analysis showed that recurrence was associated with upregulation of pathways related to cell cycle, TP53, and IL-12 among the older group, while inflammatory pathways such as IL-33 and IL-17 were upregulated in younger patients (Fig. 1J, **Supplementary Table 11**). In TRACERx tumor samples, there were overall compositional differences in the tumor transcriptome between recurrence groups among old and young patients (Figs. 1K, L, PERMANOVA, p = 0.01, p = 0.01, respectively). Among older patients, recurrence was associated with upregulation of 3,355 genes and downregulation of 2,462 genes, while among young patients, recurrence was associate with upregulation of 3,548 and downregulation of 3,198 genes (Figs. 1M, N, **Supplementary Table 10**). Pathway analysis was consistent with the NYU cohort, with more upregulated pathways associated with recurrence among older patients compared to young patients, that included cell cycle and aging associated pathways such as: Cell cycle checkpoints, DNA replication, neutrophil degranulation, stress induced senescence and SASP (Fig. 1O, **Supplementary Table 11**).

Using GSEA, we compared the transcriptome between old and young patients across three cohorts (NYU, TCGA, and TRACERx) and examined recurrence-associated gene expression signatures stratified by age. Among older patients, we observed a statistically significant overlap in recurrence-associated gene sets between NYU and TRACERx cohorts (**Supplementary Fig. 9A**; asterisk), but not between NYU and TCGA. In contrast, among younger patients, a significant overlap was detected only between TCGA and TRACERx, while comparisons involving NYU showed no significant overlap (**Supplementary Fig. 9B**). Differential gene expression analyses were repeated while adjusting for sex, smoking status and stage. Results were concordant between unadjusted and core-adjusted models among old and young age groups. Pathway-level enrichment patterns were similarly preserved after adjustment (**Supplementary Fig. 10, Supplementary Tables 12–13**). Models additionally incorporating surgical factors, treatment factors, and pathological features were incomplete, inconsistent across cohorts, and limited to a substantially smaller subset and demonstrated attenuation consistent with markedly reduced power rather than reversal of biological directionality. Therefore, core adjusted models were retained as primary analyses.

To further identify transcriptomic signatures associated with recurrence, we conducted gene co-expression network analysis using MEGENA to identify gene modules associated with recurrence among the two age groups. All modules enrichment according to GO terms are detailed in **Supplementary Table 14**. In tumor samples, we identified statistically significant (FDR ≤ 0.05) modules enriched with DEGs in recurrence vs. non-recurrence in NYU, TCGA, and TRACERx cohorts, respectively (Fig. 2A, **Supplementary Table 15**). Overall, in the older group, there were more modules enriched with dysregulated genes in recurrence vs. no recurrence compared to the young group in NYU and TCGA, but not TRACERx. Furthermore, different modules were associated with recurrence among old and young patients. For example, in NYU cohort, the modules annotated to cell cycle (M12, M169, and M155), ECM organization, and cell adhesion (M7, M155) were significantly enriched with differentially expressed genes in tumor samples of old patients with recurrence compared to non-recurrence (Fig. 2B, **Supplementary Fig. 11A)**, while M164, annotated to cell surface receptor signaling and system development, was significantly enriched with differentially expressed genes in tumor samples of old patients without recurrence (fold enrichment [FE] = 4.00, adjusted *p* = 4.41E-09,). Findings were further confirmed in the TCGA and TRACERx cohorts and found to be conserved across the cohorts. For example, M12, M169, M170, and M661, annotated to cell cycle processes and glycolysis, were enriched with upregulated genes in the recurrent group across all three cohorts. M646, annotated to the regulation of Wnt signaling pathway, and M164, system development, were also significantly enriched with differentially expressed genes in patients without recurrence across the three cohorts (Fig. 2B). Among young patients, the modules (M166, M651, M1152, and M1407) annotated to response to chemical stimulus, intracellular signal transduction, response to cytokines, apoptosis, and Th-17 cell pathways were significantly enriched with upregulated genes in the recurrence group, while modules annotated to lipid metabolic process and surfactant metabolism (M4, M104 and M435) were significantly enriched with differentially expressed genes in the non-recurrence group, which were also confirmed in both the TCGA and TRACER cohorts (Fig. 2B, **Supplementary Fig. 11B, Supplementary Tables 14–15**).

We further evaluated modules associated with recurrence using an interaction model that incorporates age as either a discrete or a continuous variable (**Supplementary Table 16**). In the NYU cohort, several modules emerged as significantly enriched in recurrence-related gene expression. M158, microtubule-based, and M8, cilia processes and movement, showed the strongest associations under both discrete and continuous age models, with highly significant enrichment and large gene overlaps. Additional modules such as M582, M1003, M559, and M152 also demonstrated consistent enrichment across both models, indicating stability of signal regardless of how age is modeled. Several of these NYU-derived modules were validated in the TCGA and TRACERx cohorts. M158 and M8, which were annotated to cilia and microtubule processes, showed significant enrichment across all three datasets in both discrete and continuous models. M582, cilia /microtubule movements and processes, and M1003 were also consistently identified in TCGA and appeared in TRACERx under the discrete model. In contrast, certain modules, such as M604, metabolism-associated pathways, and M1418 were uniquely enriched when age was modeled continuously, suggesting that modeling age as a linear variable can uncover distinct biology not captured by group comparisons. These cross-cohort and cross-model consistencies, such as modules annotated to cell cycle, metabolism, cilia and microtubule processes (M170, M12, M169, M661, M8, and M158), highlight robust and age-modulated transcriptional signatures associated with recurrence.

### NAT transcriptomic signatures associated with recurrence vary between age groups

**Figure 3:**
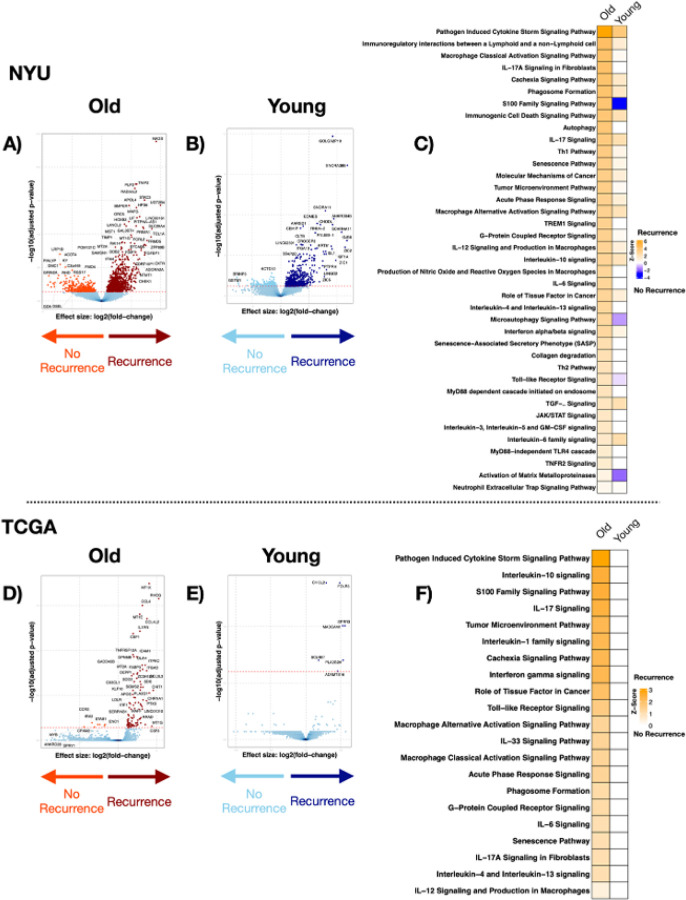
Transcriptomic signatures associated with recurrence in normal adjacent lung tissue (NAT). A, B) volcano plot of differentially expressed genes (FDR <0.2) between subjects with recurrence and without recurrence in old and young patients in the NYU cohort, respectively. C) heatmap of canonical pathway analysis based on IPA comparing recurrence vs. no recurrence groups in old and young patients within the NYU cohort. Yellow shows upregulation of pathways, and blue shows downregulation. D, E) TCGA cohort volcano plot of differentially expressed genes (FDR <0.2) between subjects with recurrence and without recurrence in old and young patients, respectively. F) TCGA cohort, heatmap of canonical pathway analysis based on IPA comparing recurrence vs. no recurrence in old and young patients. Yellow shows upregulation of pathways, and blue shows downregulation.

Among NYU NAT samples, we found no differences in beta diversity between recurrence groups among old and young patients (**Supplementary Fig. 12A, B**). However, recurrence was associated with 1191 upregulated and 350 downregulated genes within the old group and 687 upregulated and 219 downregulated genes within the young group (Fig. 3A, 3B, **Supplementary Table 8**). Importantly, IPA analyses showed that recurrence within the old group was associated with dysregulation of cancer, inflammation and aging-associated pathways, such as IL-6, IL-10, IL-17, interferon and other senescence pathways (Fig. 3C, **Supplementary Table 9)**.

We then analyzed the TCGA NAT samples. While no significant differences were found in beta diversity (**Supplementary Fig. 12C, 12D**), several genes were significantly dysregulated in old patients with recurrence (98 up- and 8 down-regulated) and were annotated to pathways noted in NYU NAT samples such as IL-1, IL-6, IL-10, IL-17, Interferon, and Senescence. In contrast, we found only six significantly dysregulated genes among young patients with recurrence, limiting our pathway analysis ability (Fig. 3D–3F, **Supplementary Tables 10,11**). These findings suggest that age associated differences in recurrence are more readily seen in NAT samples than tumor samples. In summary: 1) more pathways that are associated with recurrence are upregulated in old patients than in young, and 2) these pathways include those activated in aging such as inflammation, signaling dysfunction, and senescence related pathways.

Using GSEA we examined recurrence associated gene expression signatures overlap between cohorts among age groups and identified 29 shared genes between NYU and TCGA among old patients, and 0 shared genes between the two cohorts among young patients (**Supplementary Fig. 13**), none of them achieved statistical significance. As with tumor samples, analyses were adjusted for confounders demonstrating concordant results between unadjusted and core-adjusted models across NAT samples and among age groups (old and young) with preserved pathway-level enrichment patterns after adjustment (**Supplementary Fig. 14, Supplementary Tables 17–18**). As with tumor samples, models incorporating surgical factors, treatment factors, and pathological features were incomplete, inconsistent across cohorts, and limited to a substantially smaller subset and. Therefore, core adjusted models were retained as primary analyses.

Gene co-expression network analysis of NAT samples showed that among old patients, 9 statistically significant modules were enriched with DEGs when comparing recurrence vs. no recurrence, while 13 modules were enriched among young patients. In comparison, in TCGA, 12 and 13 modules were significantly associated with recurrence among old and young patients, respectively. Furthermore, different modules were identified among old patients and young patients in both cohorts (Fig. 4A, **Supplementary Table 19**). Among the top modules that were enriched with DEGs associated with recurrence in the old patients include modules M166, cellular response to chemical stimulus, IL/cytokine signaling, M151, immune system, and M155, ECM organization. Among young patients, the modules annotated to gene expression and transcription (such as M604, M1274) were enriched with DEGs among patients with recurrence, while module M164 annotated to calcium signaling and matrisome/ECM were enriched with DEGs among patients without recurrence (Fig. 4B, **Supplementary Fig. 15, Supplementary Table 14,19**). Some modules were conserved in NAT samples from both the NYU and TCGA cohorts. For example, modules annotated to respond to chemical stimulus, intracellular signal transduction, response to cytokines, IL-17 cell pathways modules (M166, M651, M1152, and M1407) that were upregulated in older patients with recurrence were conserved across the NYU and TCGA cohorts (Fig. 4B).

In age interaction models that incorporate age as a discrete or a continuous variable, we identified modules associated with recurrence and age group. For instance, using discrete age model, we found that significant association with modules M151, M6, and M535, which were enriched with genes annotated to cytokines/cytokines interaction in NAT samples from the NYU cohort (**Supplementary Table 20**). These findings were further confirmed in the TCGA cohort, with modules annotated to cytokine signaling and interactions identified by the discrete age model as associated with recurrence (such as M155, M1407, M1152, M651). Continuous age interaction model identified only M5 in NYU cohort as associated with age and recurrence, while multiple modules were identified in TCGA including M7, M155, M568, M164, and M990, which were concordant with the discrete model results. These findings highlight cross-cohort and cross-model consistencies and show aging related differences influencing recurrence risk between tumor and NAT tissue.

## Discussion

In this study of stage I lung adenocarcinoma, gene expression profiles from tumor and tumor-adjacent normal lung showed that the lung transcriptome from older patients were dominated by genes associated with inflammation, signaling dysfunction, and senescence. Additionally, in older patients with recurrence, we observed upregulation of genes related to cancer, inflammation, and senescence, such as DNA replication, glycolysis, IL-17, IL-6, and interferon signaling. In contrast, fewer genes were found to be differentially regulated between the recurrence vs. no recurrence group among young patients. Our findings were highly conserved among three independent cohorts, and to our knowledge, this is the first study to show aging-related transcriptomic differences in early-stage NSCLC. These findings suggest that biological aging contributes to the recurrence of early-stage lung cancer following surgical resection and may in fact be a key player in lung cancer progression.

Recent publications have examined the association between transcriptomics and aging in NSCLC. Zhou et al. identified differentially expressed genes between LUAD patients > 70 years and those ≤ 70 years and showed that these genes annotate to pathways related to carcinogenesis^33^. Pham-Danis et al., analyzed public databases of gene expression and found that pathways such as inflammatory response, TNF-α, interferon-γ, and ECM organization were upregulated among old vs. young patients with NSCLC^34^. We observed similar upregulated pathways associated with cell cycle, carcinogenesis, and persistent inflammation when we compared old vs. young patients. However, these studies suffer from heterogeneity in cancer histology, stages, and treatments, that may confound results. Furthermore, these studies focus on mortality as a clinical outcome of interest which can be due to a multitude of causes. We focused on recurrence as a clinical outcome as it may reveal mechanisms related to carcinogenesis among early-stage NSCLC. Our study extends previous findings to reveal an association of signatures not only with age, but also with cancer recurrence.

Differences in gene expression between old and young patients are not limited to lung cancer, as several pan-cancer studies have reported age-related gene expression changes in tumors that are associated with numerous biological processes, such as ECM organization, signaling pathways, and immune processes^5,35,36^. In a pan-cancer study conducted on TCGA, patient age was significantly associated with enrichment for interferon-γ, TGF-β and canonical WNT pathways^37^. These findings are consistent with our data in NAT samples, that show upregulation of such inflammatory and immune-stimulatory signaling in the tumor microenvironment of old patients with recurrence. As these results are derived from bulk RNA-seq, they likely incorporate changes from the aging tissue microenvironment, which contribute to tumor progression via increased inflammation and senescence. These processes are among the hallmarks of cellular aging that lead to gradual deterioration of tissue function and increased vulnerability to disease, and lead to worse outcomes among older patients^8,38^. Importantly, the mechanisms linking aging to recurrence may reflect both cell-autonomous changes within tumor cells (e.g., impaired DNA repair, genomic changes) and non-cell-autonomous influences driven by the aged microenvironment, including senescence, altered cytokine secretion, and immune remodeling^6–8^. This includes elements of immunosenescence—diminished antigen presentation, and chronic low-grade inflammation—which may further weaken anti-tumor immunity in older patients and potentially affect response to therapy^6,39^. Our findings suggest that aging plays a role in pro-tumor activity, promotes tumor progression, and might contributes to poor outcomes among older patients with cancer. However, our findings are observatory and not causal, and future mechanistic studies aiming to examine the role of aging signatures mechanistic and therapeutic potential are needed.

We have previously showed that distinct transcriptome signatures exist in both tumor and NAT in NSCLC^40,41^. Our current study shows tissue-specific differences and revealed an additional layer of age-related divergence. We noted differences in gene expression directionality between tumor and NAT samples; for example, in tumor samples module M164,annotated to ECM, was enriched with expressed genes associated with no recurrence among old patients (Fig. 2D), while in NAT samples, the same module was enriched with genes associated with no recurrence among young patients (Fig. 4D), and no enrichment was found in old patients. This inversion in module directionality highlights that aging modifies recurrence-associated transcriptional programs differently across tissues. These results align with prior evidence that aging alters the tumor microenvironment and host tissue responses, particularly through extracellular matrix and immune pathways^7,14^. In younger NAT, enrichment of these signatures may indicate protective or regenerative programs that diminish with age, while in tumors they may reflect age-driven stromal remodeling that influences progression. Taken together, our findings suggest that recurrence risk is shaped by the combined effect of tissue context and aging, and that regulatory pathways protective in one compartment or age group may not hold the same role in another. These contradictory observations may explain the variability in clinical outcomes between younger and older patients.

There are several limitations to our study. First, we note variabilities among the cohorts. In general, TCGA and TRACERx patients were younger than the NYU cohort, limiting the number of old patients and subgroup analyses. We chose to stratify patients using the median age cut-off (70 years) to distinguish old and young patients. While there is no clear cutoff for the definition of old and young patients, the cutoff of 70 aligns with the median age of lung cancer diagnosis (71 years of age)^18^. There are other inherent differences between the cohorts including in demographics, environmental exposures, and in data generation (e.g., tissue handling, RNA isolation, library construction, sequencing, etc.). We noticed differences in survival and recurrence using cutoffs between the two groups. Importantly, when age was modeled as a continuous variable, recurrence-related signals strengthened with increasing age, supporting the robustness of our findings even beyond 80 years old. This is notable given the USPSTF lung cancer screening recommendations up to age 80^42^, as our data suggest that older patients may still face a higher risk of poor outcomes despite cutoffs of lung cancer screening. Second, there were relatively small number of recurrence events in our NYU cohort (n = 27) and inconsistencies in some findings between the NYU and TCGA cohorts, limiting our ability to validate the data, particularly among the NAT samples. Differences may be related to several factors, including a low number of NAT samples among the TCGA cohort (none were available in TRACERx), demographics differences, methodological differences in sample processing and sequencing approaches, and TCGA patients were generally younger than our NYU cohort. Given these limitations, our findings are exploratory and larger cohorts with consistent design and approaches are required to validate our findings and expand our understanding of aging role in lung cancer prognosis. Third, our cohorts lack consistent data on comorbidities and confounders data. Data on chronic lung comorbidities, cachexia, weight loss, frailty, surgical factors, tumor biology and pathological features were incomplete and could not be systematically incorporated; therefore, observed aging associated signatures may partially reflect underlying pulmonary disease burden, and transcriptomic changes may reflect broader vulnerability to adverse outcomes beyond cancer. However, this study focused on cancer specific recurrence, and it was not designed to comprehensively model all determinates of recurrence but rather to test if transcriptomic signatures are observable despite clinical heterogeneity. Future investigations should evaluate other causes of mortality. Lastly, we did not elucidate all potential mechanisms underlying the associations between aging, recurrence, and immune cells, which requires further experimental validation of the tumor microenvironment.

In conclusion, we identified transcriptome signatures of aging in an early-stage lung adenocarcinoma population characterized by cancer- and inflammation-related alterations associated with post-surgical removal cancer recurrence. These findings highlight the potential value of aging-associated molecular signatures for improving risk stratification in early-stage disease. However, given the observational nature of this study, these associations should not be interpreted as causal mechanisms. Rather, the identified pathways, including senescence and cytokine associated programs such as IL-6, and IL-17 signaling, provide a biological framework for future mechanistic and cell-type-resolved studies aiming at clarifying how aging-related host programs interact with lung cancer biology.

## Supplementary Material

Supplementary Files

This is a list of supplementary files associated with this preprint. Click to download.

- Supp.Tables.aging.lung.cancer0226FD.xlsx
- analysisbyageprogressreportv16copysupp.pdf
- SupplementalFiguresLegends.pdf

## Figures and Tables

**Table 1:** NYU internal cohort patients’ demographical and clinical characteristics

	Total	Old	Young	p-value
	(N = 126)	(N = 60)	(N = 66)	
**Age (Median [IQR])**	70.0 [63.0, 77.0]	77.5 [74.0, 82.0]	63.5 [59.0, 67.0]	**0.001**
**Sex**				
Male	38 (30.2%)	21 (35.0%)	17 (25.8%)	0.35
Female	88 (69.8%)	39 (65.0%)	49 (74.2%)	
**Ethnicity**				0.78
White	101 (80.2%)	50 (83.3%)	51 (77.3%)	
Black / African American	3 (2.4%)	1 (1.7%)	2 (3.0%)	
Hispanic / Latino	2 (1.6%)	0 (0%)	2 (3.0%)	
Asian	15 (11.9%)	7 (11.7%)	8 (12.1%)	
Other	5 (4.0%)	2 (3.4%)	3 (4.5%)	
**Smoking**				0.62
Current / Past	93 (73.8%)	46 (76.7%)	47 (71.2%)	
Non-Smoker	33 (26.2%)	14 (23.3%)	19 (28.8%)	
**Pack Years (Median [IQR])**	20.0 [0, 40.0]	25.0 [5.00, 42.9]	11.0 [0, 32.3]	**0.003**
**Stage**				0.08
IA	94 (74.6%)	40 (66.7%)	54 (81.8%)	
IB	32 (25.4%)	20 (33.3%)	12 (18.2%)	
**Tumor Size, cm (Median [IQR])**	1.80 [1.50, 2.50]	2.10 [1.50, 2.73]	1.80 [1.40, 2.38]	**0.02**
**Recurrence**	27 (21.4%)	14 (23.3%)	13 (19.7%)	0.78
**Recurrence Type**				0.55
Locoregional	14 (11.1%)	6 (10.0%)	8 (12.1%)	
Systemic	13 (10.3%)	8 (13.3%)	5 (7.6%)	
**5-Years Mortality**	15 (11.9%)	12 (20.0%)	3 (4.5%)	**0.01**

Data expressed as n (%) or median [interquartile range].

p-values denotes chi square and Kruskal-Wallis for categorical and continuous variables, respectively.

## Data Availability

Raw data of the NYU cohort from the RNA sequencing is available at Sequence Read Archive PRJNA987649. TRACERx annotated tables are available at https://doi.org/10.5281/zenodo.7603386^19^. The annotated tables and analytical codes used for the analyses presented in the current manuscript are available at https://github.com/segalmicrobiomelab/aging.lung.transcriptome.lung.cancer.git
